# Brassinolide improves the tolerance of *Malus hupehensis* to alkaline stress

**DOI:** 10.3389/fpls.2022.1032646

**Published:** 2022-11-24

**Authors:** Zhijuan Sun, Yawen Zou, Cheng Xie, Lei Han, Xiaodong Zheng, Yike Tian, Changqing Ma, Xiaoli Liu, Caihong Wang

**Affiliations:** ^1^ College of Life Science, Qingdao Agricultural University, Qingdao, China; ^2^ College of Horticulture, Qingdao Agricultural University, Qingdao, China; ^3^ Qingdao Key Laboratory of Genetic Improvement and Breeding in Horticulture Plants, Qingdao, China

**Keywords:** brassinolide, *Malus hupehensis*, alkaline stress, rhizosphere pH, oxidative damage

## Abstract

*Malus hupehensis* is one of the most widely used apple rootstocks in china but is severely damaged by alkaline soil. Alkaline stress can cause more serious harmful effects on apple plants than salt stress because it also induces high pH stress except for ion toxicity, osmotic stress, and oxidative damage. Brassinolide (BL) plays important roles in plant responses to salt stress. However, its role and function mechanism in apple plants in response to alkaline stress has never been reported. This study showed that applying exogenous 0.2 mg/L BL significantly enhanced the resistance of *M. hupehensis* seedlings to alkaline stress. The main functional mechanisms were also explored. First, exogenous BL could decrease the rhizosphere pH and promote Ca^2+^ and Mg^2+^ absorption by regulating malic acid and citric acid contents and increasing H^+^ excretion. Second, exogenous BL could alleviate ion toxicity caused by alkaline stress through enhancing Na^+^ efflux and inhibiting K^+^ expel and vacuole compartmentalization. Last, exogenous BL could balance osmotic stress by accumulating proline and reduce oxidative damage through increasing the activities of antioxidant enzymes and antioxidants contents. This study provides an important theoretical basis for further analyzing the mechanism of exogenous BL in improving alkaline tolerance of apple plants.

## Introduction

Soil salinization seriously restricts the sustainable development of agricultural production around the world (Hu et al., 2016; Liang et al., 2017; Christian et al., 2018). About 20% of the global arable land is affected by salinization, which will continue to expand with global warming and excessive application of pesticides and fertilizers (Xia et al., 2019; Negi et al., 2021). Apple (*Malus domestica* Borkh.) is one of the most valuable horticultural fruit crops and widely cultivated in the world (Ma et al., 2019). However, large areas of salinization soil exist in main apple producing areas, resulting in yellow leaves and weakening the growth of the fruit trees, which seriously affect the production and quality of apples (An et al., 2018; Su et al., 2020). Therefore, improving the tolerance of fruit trees to saline-alkali stress is of great significance for effectively utilizing saline-alkali land and giving full play to its economic and ecological effect.

Saline-alkali stress in natural environment is usually accompanied by neutral salt stress (caused by NaCl) and alkaline stress (caused by NaHCO_3_ and Na_2_CO_3_) (Liu et al., 2015). Plants have different response mechanisms to neutral salt stress and alkaline stress (Sharma et al., 2016), and the latter causes a more complex and significant damage to plants than the former (Wang T. et al., 2015; Gong et al., 2017). Under alkaline stress, in addition to osmotic stress and ion poisoning, the roots of the fruit trees suffer from damage caused by high pH stress, thereby reducing the absorption of trace elements (such as Ca^2+^, Mg^2+^, Fe^2+^ and Mn^2+^). This phenomenon causes the symptoms of element deficiency, disturbs the acid-base balance, and affects the quality of the fruit (Fan et al., 2021). Moreover, osmotic stress caused by alkaline stress could harm the photosynthetic system of plants, which usually affects the photosynthetic rate and fluorescence parameters of chlorophyll. Besides the direct damage to plants, alkaline stress could trigger oxidative damage indirectly, which result in the excessive accumulation of reactive oxygen species (ROS), leading to the destruction of plant cell membranes, impairment of vital biological processes, and acceleration of plant death. A number of previous studies focused on physiological and biochemical responses of plants to neutral salt stress. Nevertheless, the resistance mechanism to alkaline stress remains unclear in fruit trees.

In long-term struggle with alkaline stress, plants have evolved their own physiological and molecular mechanisms to adapt this situation (Mao et al., 2017; Hu et al., 2018; Chen et al., 2019). Plants can regulate the ion balance by expelling Na^+^ and absorbing K^+^ to maintain the cytoplasmic Na^+^/K^+^. Antioxidant enzymes, including superoxide dismutase (SOD), peroxidase (POD), catalase (CAT) and peroxidase (APX), in plants’ defense systems can remove ROS to reduce oxidative damage (Tofighi et al., 2017; Wu et al., 2017). Meanwhile, ascorbic acid (AsA) and glutathione (GSH) are important non-enzymatic antioxidants, which play a crucial role in quenching the ROS and protecting plants from damaging effects of highly oxidizing ROS (Wang et al., 2019; Li et al., 2022). In addition, plants can regulate osmotic potential and ion balance by increasing the concentrations of osmolytes (e.g., proline, glycine, betaine, soluble sugar, and soluble protein). Under high pH stress, plants regulate the rhizosphere pH primarily by regulating the proton pump (H^+^-ATP enzyme) activity and the secretion of organic acids (Zhan et al., 2019).

Plant hormones play an important role in plant growth and development and response to environmental stress (Ryu and Cho, 2015). Exogenous application of plant growth regulators is one of the effective ways to improve the salt and alkali resistance of crops (Shahzad et al., 2018; Su et al., 2020). Multiple plant hormones, such as abscisic acid, melatonin, and jasmonic acid, play key roles in plants’ response to salt stress (Zhu, 2016; Wu et al., 2019; Zhao et al., 2021). Brassinosteroids (BRs) are sterol hormones that regulate vegetative growth and reproductive growth in plants (Xiong et al., 2022). Exogenous analog brassinolide (BL) is recognized as a highly efficient, universal, and non-toxic regulator of plant growth, which can significantly increase the plant photosynthesis efficiency and promote nutrient growth at low concentrations (Dong et al., 2017; Nawaz et al., 2017; Tofighi et al., 2017). Exogenous application of BL can also improve the cold resistance, drought resistance, and salt resistance of crops (Sharma et al., 2013; Jia et al., 2021). BL promotes nutrient absorption and metabolism in plant growth by reducing the accumulation of toxic ions and oxidative damage, and plays a positive role in abiotic stress tolerance (Li et al., 2015). For instance, exogenous BL application improved the drought tolerance through modulation of enzymatic antioxidants and leaf gas exchange in maize (Li et al., 2020). In cucumber plants, exogenous BL application alleviated Ca(NO_3_)_2_ stress by regulating mineral nutrients uptake and distribution (Yuan et al., 2015). However, the effect of BL on apple plants growth and the underlying mechanism under alkaline stress remains unclear.

To investigate the role and mechanism of BL on *Malus hupehensis* seedlings under alkaline stress, our study analyzed the its function from four aspects: rhizosphere pH balance, ion homeostasis, osmotic regulation, and antioxidant system. Moreover, the expression of alkaline-responding genes was detected under alkaline stress and exogenous BL treatment by qPCR. This study provides an important theoretical basis for further analyzing the mechanism of exogenous BL in improving alkaline tolerance of apple plants by focusing on roots.

## Materials and methods

### Plant materials and growth conditions

Seeds of *M. hupehensis* after cold stratification were sown in a 50-hole tray containing seedling substrate [nutrient soil (65% fertile garden soil, 10% fine sand, 25% burning soil, and 0.4% calcium magnesium phosphate fertilizer) and vermiculite with the ratio of 1:1]. They were cultivated in a greenhouse under the controlled condition of photoperiod (16/8 h day/night), light intensity (100 μmol·m^-2^·s^-1^), humidity (60%-65%), and temperature (25°C). When the apple seedlings developed to six leaves, they were transplanted into plastic pots (one seedling per pot) with dimensions of 7 cm × 7 cm × 10 cm (length, width and height) and watered with Hoagland’s nutrient solution (pH = 5.9) every 3 days. After 7 days, seedlings with similar growth status were selected for alkaline stress and exogenous BL treatment.

### Alkaline stress and exogenous BL treatment

A total of 120 apple seedlings were randomly divided into three groups. The control group was irrigated with Hoagland’s nutrient solution (group I). The alkaline treatment group (group II) was irrigated with Hoagland’s nutrient solution containing 80 mM Na_2_CO_3_:NaHCO_3_ = 1:1 (pH = 8.3) every 3 days. In group III, except for the same alkaline treatment [Hoagland’s nutrient solution containing 80 mM Na_2_CO_3_:NaHCO_3_ = 1:1 (pH = 8.3)] with group II, the apple seedlings were also irrigated and sprayed with 0.2 mg/L BL (Solarbio, Beijing, China) every 3 days. After 15 days of alkaline stress and BL treatment, the phenotype of the apple seedlings was recorded. Wilting rate, plant height, fresh weight (FW), dry weight (DW), chlorophyll content, and photosynthetic rate were measured as described by Zheng et al. (2021). Total root length and fibrous root number were analyzed by root scanner (Epson, Beijing, China). Each experiment was independently repeated three times.

### Roots pH staining and determination of organic acid contents

Rhizosphere pH staining was conducted according to Zhao Q. et al. (2016). The roots of apple seedlings under alkaline stress and exogenous BL treatment were placed in culture medium containing 0.01% bromocresol purple (Solarbio, Beijing, China), 0.2 mM CaSO_4_, and 0.7% agar (pH = 6.5) for 24 h. Bromocresol purple was used as an acid-base indicator, and its pH change range was 5.2 (yellow)-6.8 (purple). Acidification was indicated by yellow colour around the apple roots. The color presents the accurate pH value.

A total of 0.5 g of roots from each treatment were used to detect the contents of organic acids, including malic acid and citric acid, as described by Li T. et al. (2021). Each experiment was independently repeated three times.

### Determination of ROS Level, antioxidant enzyme activities and antioxidants contents

Fifteen apple seedlings were randomly selected from each group for ROS detection. The ROS level in the roots and H_2_O_2_ and O_2_·^−^ staining in leaves were detected as described by Zheng et al. (2017). The content of malondialdehyde (MDA) was determined by thiobarbituric acid (TBA) as described by Alam et al. (2019).

Each 0.1 g of apple seedling roots or leaves were separately weighed to detect antioxidant enzyme activities and antioxidants contents. The apple seedlings roots were ground into homogenates on ice and then centrifuged at 12 000 rpm for 10 min at 4°C. For the assay of activity of SOD, the reaction mixture comprising enzyme extract (100 µL), phosphate buffer (100 mM, pH 7.4), riboflavin (50 µM), EDTA (1.0 mM), methionine (10 mM), and NBT (75 µM) was kept for 15 min under fluorescent light. The optical density (OD) was noted at 560 nm (Alam et al., 2019). POD activity was estimated using the guaiacol colorimetric at 470 nm for 1 min, as described by Zheng et al. (2017). The activity of CAT was detected by detecting the reduction of H_2_O_2_ at 510 nm for 5 min by the method of Alam et al. (2019). APX activity was assayed following a reduction in absorbance of the mixture containing hydrogen peroxide and ascorbic acid at 290 nm for 3 min as described by Alam et al. (2019).

Each 0.1 g of apple seedling roots were ground into homogenates in liquid nitrogen and then centrifuged at 12 000 rpm for 10 min at 4°C. GSSG, GSH, AsA, and DHA contents were measured using visible photometry as described previously (Li et al., 2018; Song et al., 2018; Ji et al., 2019). Each experiment was independently repeated three times.

### Determination of electrolyte leakage and osmolyte content

Ten seedlings were randomly selected from each group after treatment for 15 days to detect electrolyte leakage as described previously (Ahmad et al., 2016). Firstly, the electrical conductivity (ECa) of the 10 leaf disks submerged was measured. Secondly, the leaf disks were put in test tubes and incubated at 55°C for 25 min, and the electrical conductivity (ECb) was measured. Finally, the test tubes were boiled at 100°C for 10 min, and the electrical conductivity (ECc) was determined. Electrolyte leakage was calculated using the following formula: electrolyte leakage (%) = (ECb − ECa)/ECc × 100. Both the biological and technical duplications of each experiment were repeated three times.

0.5 g of roots from each group were used to detect osmolytes including proline and soluble sugar. The roots were ground in 5 mL of pre-cooled extracted buffer and centrifuged (12,000 rpm) at 4°C for 10 min. The supernatant was used for proline and soluble sugar content assays as described by Li T. et al. (2021). Each experiment was independently repeated three times.

### Quantification of mineral elements

Fifteen apple seedlings were randomly selected from each group and cleaned with distilled water after 15 days of treatment. The samples were dehydrated at 105°C for 30 min and then baked in an oven at 80°C for 72 h. A total of 0.5 g of the dried roots were ground into powder and mixed with 10 mL of HNO_3_ and 2 mL of HClO_4_ to digestion. The solution was added with deionized water and diluted to 25 mL. The contents of Na^+^, K^+^, Ca^2+^, Fe^2+^, Mg^2+^, and other mineral ions were determined by inductively coupled plasma atomic emission spectrometry (PerkinElmer, Waltham, USA), as described by Li T. et al. (2021). Each experiment was independently repeated three times.

### RNA extraction and quantitative real-time PCR analysis

After alkaline stress and exogenous BL treatments, the total RNA of different groups was extracted using the RNAprep pure Plant Plus Kit (Tiangen, Beijing, China). Inverse transcription and qPCR assay were conducted as described by Zheng et al. (2021). *MhActin* (accession number: MDP0000774288) was used as internal control. Primer sequences for qPCR were designed according to the coding sequence of *MhAHAs*, *MhNHXs*, *MhBZRs*, *MhMATE1*, *MhALMT1*, *MhSOS1*, *MhCHX15*, and *MhSKOR* by using Primer 5 software and checked using BLAST search in the apple genomic database. The primer sequences are shown in **Table S1**. Each experiment was independently repeated three times.

### Experimental design and statistical analysis

All experiments were repeated three times. Data were analyzed by ANOVA followed by Fisher’s least-significance difference or Student’s *t*-test analysis. Statistically significant differences were indicated by *P*-value < 0.05. Statistical computations were conducted using SPSS (IBM, Armonk, USA).

## Results

### Effects of exogenous BL on the aboveground phenotype of apple seedlings under alkaline stress

**Figure 1:**
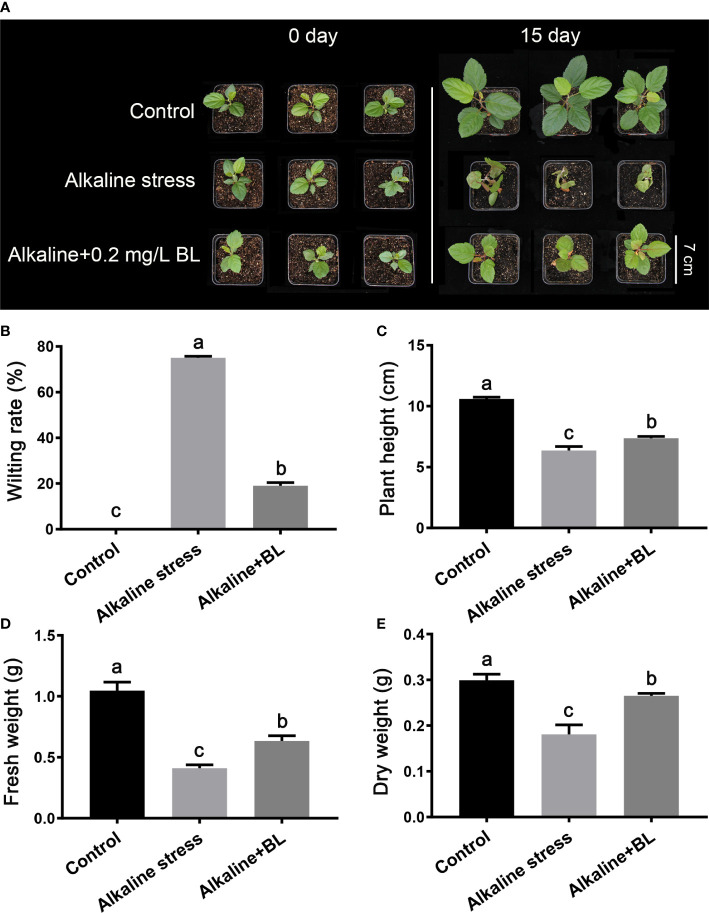
The aboveground phenotypes of *M. hupehensis* seedlings treated with alkaline stress and 0.2 mg/L BL. **(A)** The phenotypes of *M. hupehensis* seedlings treated with alkaline stress and BL at day 0 and day 15. The scale bar represents 7.0 cm. Effects of exogenous BL on wilting rate **(B)**, plant height **(C)**, fresh weight **(D)**, and dry weight **(E)** of the seedlings after alkaline stress for 15 days. Data represent the mean ± SD of triplicate experiments. Different lowercase letters indicate significant differences according to Fisher’s least significant difference (*P* < 0.05).

As shown in **Figure 1A**, the apple seedlings were wilted and seriously damaged by alkaline stress, and the wilting rate was as high as 75%. After application of 0.2 mg/L BL for 15 days, the growth vigor of exogenous BL-treated seedlings was better than that of the alkaline-stressed seedlings. The wilting rate also decreased to 19% (**Figure 1A, B**). In addition, the plant height, fresh weight, and dry weight were detected. The plant height under alkaline stress was only 6.4 cm, which was 4.2 cm shorter than that of the control, while it only reduced by 3.2 cm after application of exogenous BL (**Figure 1C**). After 15 days of alkaline stress, the fresh weight and dry weight were significantly lower than those in the control group. However, after 15 days of treatment with exogenous BL, the fresh weight and dry weight increased by 54.5% and 47.2%, respectively, compared with those under alkaline stress (**Figure 1D, E**). These results indicated that alkaline stress seriously inhibited the aboveground part growth of the apple seedlings, and the treatment of exogenous BL significantly alleviated this damage.

### Effects of exogenous BL on chlorophyll content and photosynthetic rate under alkaline stress

**Figure 2:**
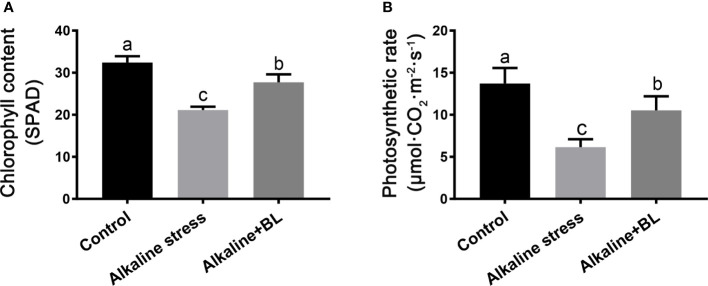
Effects of exogenous BL treatment on chlorophyll content **(A)**, photosynthesis rate **(B)** of *M. hupehensis* seedlings under alkaline stress. The data represent the mean ± SD of three biological replicates. Different lowercase letters indicate significant differences according to Fisher’s least significant difference (*P* < 0.05).

Alkaline stress caused plant wilting, and exogenous BL alleviated the chlorosis of the apple seedlings under alkaline stress. Chlorophyll content and photosynthetic rate were measured to explore the physiological mechanism. As shown in **Figure 2A**, the chlorophyll content in the apple seedlings under alkaline stress was only 21.1 SPAD, which reduced a third compared with that in the control group (32.4 SPAD). When exogenous BL was applied, the chlorophyll content significantly increased to 27.7 SPAD. The variation in photosynthetic rate was similar to that in chlorophyll content. The photosynthetic rate was significantly inhibited under alkaline stress but increased after application of exogenous BL. Under alkaline stress, the photosynthetic rate decreased significantly but recovered when exogenous BL was applied (**Figure 2B**). These results suggested that exogenous BL protected the chlorophyll level and photosynthetic system from alkaline stress.

### Effects of exogenous BL on the underground phenotype of apple seedlings under alkaline stress

**Figure 3:**
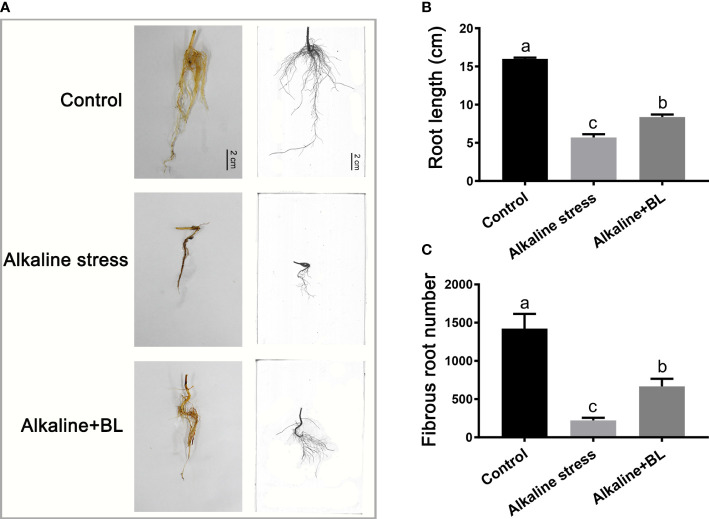
The underground phenotypes of *M. hupehensis* seedlings treated with alkaline stress and exogenous BL. **(A)** The roots phenotypes of the seedlings treated with alkaline stress and BL at day 15. The scale bar represents 2.0 cm. Effects of exogenous BL on root length **(B)** and fibrous root number **(C)** of the seedlings after alkaline stress for 15 days. The data represent the mean ± SD of three biological replicates. Different lowercase letters indicate significant differences according to Fisher’s least significant difference (*P* < 0.05).

For the underground part, the root growth of the apple seedlings was seriously inhibited under alkaline stress, but the inhibitory effect was alleviated after application of exogenous BL (**Figure 3A**). The root length of the apple seedlings was only 5.69 cm under alkaline stress, which was 10.30 cm shorter than that of the control group, but it increased significantly to 8.37 cm after exogenous BL was applied (**Figure 3B**). Similar to root length, the fibrous root number under alkaline stress was decreased significantly compared with the control group and increased by about threefold after exogenous application of BL (**Figure 3C**). The above results indicated that exogenous BL treatment significantly alleviated the root damage caused by alkaline stress.

### Effects of exogenous BL on rhizosphere pH and organic acid contents under alkaline stress

**Figure 4:**
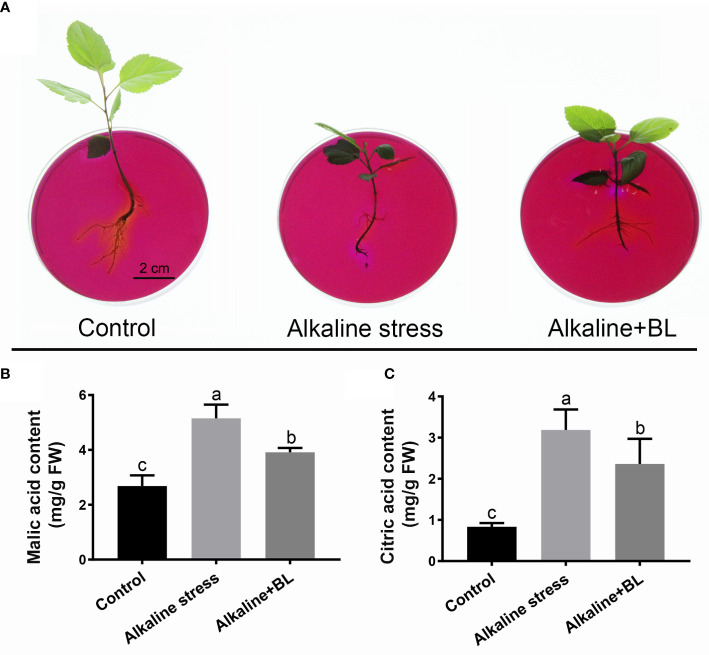
Effects of exogenous BL on rhizosphere pH and organic acid contents of *M. hupehensis* under alkaline stress. **(A)** Effects of alkaline stress and exogenous BL on rhizosphere pH of the seedlings by bromocresol violet staining. The scale bar represents 2.0 cm. Effects of alkaline stress and exogenous BL on malic acid content **(B)** and citric acid content **(C)** of the seedlings at day 15. The data represent the mean ± SD of three biological replicates. Different lowercase letters indicate significant differences according to Fisher’s least significant difference (*P* < 0.05).

Alkaline stress would cause high pH stress to the roots. A medium containing the pH indicator bromocresol purple could be effective in reflecting rhizosphere pH. As shown in **Figure 4A**, the surrounding of the roots turned purple under alkaline stress, but it returned yellow after application of exogenous BL, meanwhile, the control roots showed yellow color. Moreover, the contents of malic acid and citric acid under alkaline stress increased significantly, but decreased respectively after application of exogenous BL (**Figure 4B, C**). These results demonstrated that exogenous BL alleviated the high pH stress caused by alkaline stress by regulating organic acid contents.

### Effects of exogenous BL on the oxidative damage under alkaline stress

**Figure 5:**
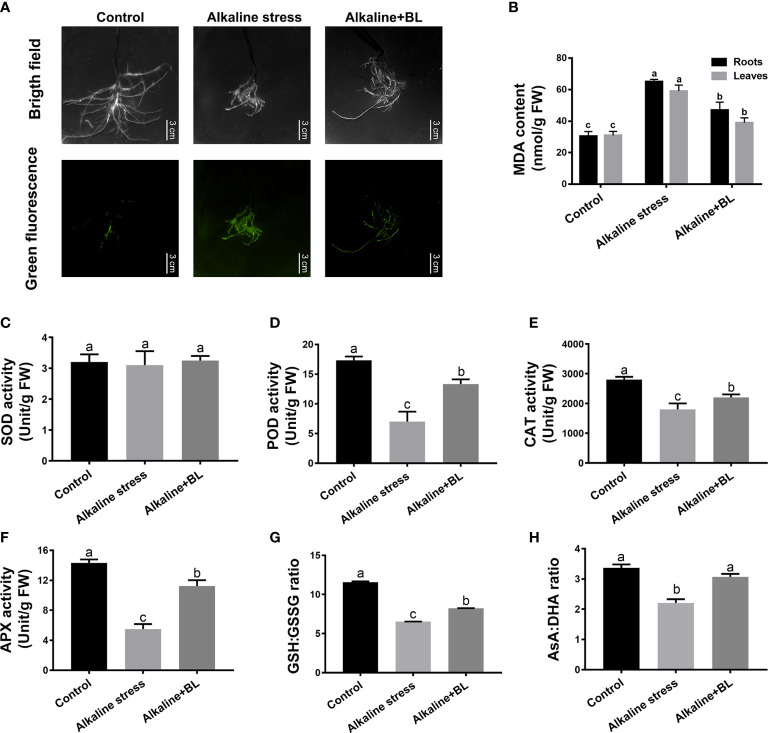
Effects of exogenous BL on the oxidative damage, antioxidant enzyme activities and antioxidants contents of *M. hupehensis* seedlings roots under alkaline stress. **(A)** Effects of alkaline stress and exogenous BL on the ROS level in roots. The scale bar represents 3.0 cm. Effects of exogenous BL treatment on MDA content **(B)**, SOD activity **(C)**, POD activity **(D)**, CAT activity **(E)**, APX activity **(F)**, GSH : GSSG ratio **(G)** and AsA : DHA ratio **(H)** under alkaline stress. The data represent the mean ± SD of three biological replicates. Different lowercase letters indicate significant differences according to Fisher’s least significant difference (*P* < 0.05).

The fluorescence staining results revealed that alkaline stress significantly elevated the ROS levels in the roots of apple seedlings, but the levels were significantly decreased when exogenous BL was applied (**Figure 5A**). Consistently, the MDA contents in the roots and leaves under alkaline stress were more than twice that in the control group, but significantly decreased after the application of exogenous BL (**Figure 5B**). The activities of SOD, POD, CAT and APX in roots were detected. As shown in **Figure 5C**, the SOD activity in roots had no significant change under alkaline stress and exogenous BL treatment. In contrast to SOD, the POD, CAT and APX activities in roots showed a significant decline under alkaline stress, nevertheless, enhanced after exogenous application of BL. The POD, CAT and APX activities under alkaline stress were significantly lower than those in the control group, but increased significantly when exogenous BL was applied (**Figures 5D–F**). Moreover, the ratios of GSH/GSSG and AsA/DHA showed a significant decline under alkaline stress, but increased evidently after application of exogenous BL (**Figures 5G, H**).

Furthermore, the above indicators of oxidative damage were also determined on leaves. The leaves exhibited a notably increased H_2_O_2_ level relative to the WT control under alkaline stress, but a decreasing trend after application of exogenous BL. However, the level of O_2_·^−^ in leaves had no significant change under alkaline stress and exogenous BL treatment (**Figure S1A**). The activities of SOD, POD and CAT in leaves were also detected. Similar to the roots, the SOD activity in leaves had no significant change under alkaline stress and exogenous BL treatment. In contrast to SOD, the POD and CAT activities in leaves were also decreased significantly under alkaline stress, but increased when exogenous BL was applied (**Figure S1B-D**). The above results suggested that exogenous BL could alleviate oxidative damage by improving the activities of the antioxidant enzymes (POD, CAT, and APX) and increasing the ratios of GSH/GSSG and AsA/DHA in the non-enzymatic antioxidant protection system of apples.

### Effects of exogenous BL on the electrolyte leakage and osmolytes of apple seedling roots under alkaline stress

**Figure 6:**
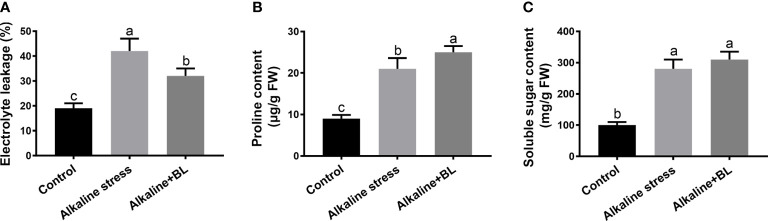
Effects of exogenous BL treatment on electrolyte leakage **(A)**, proline content **(B)**, and soluble protein content **(C)** under alkaline stress. The data represent the mean ± SD of three biological replicates. Different lowercase letters indicate significant differences according to Fisher’s least significant difference (*P* < 0.05).

As shown in **Figure 6A**, after alkaline stress, the electrolyte leakage increased significantly from 19% to 42% compared with that in the control group but decreased to as low as 32% when exogenous BL was applied. In addition, we measured the contents of osmolytes including proline and soluble sugar. Both of them were significantly induced by alkaline stress. However, the proline content was significantly increased, while the soluble sugar content had no remarkable change when exogenous BL was applied compared with that under alkaline stress group (**Figure 6B, C**). These results indicated that exogenous BL balanced the osmotic stress by accumulating proline.

### Effects of exogenous BL on the mineral elements of apple seedling roots under alkaline stress

**Figure 7:**
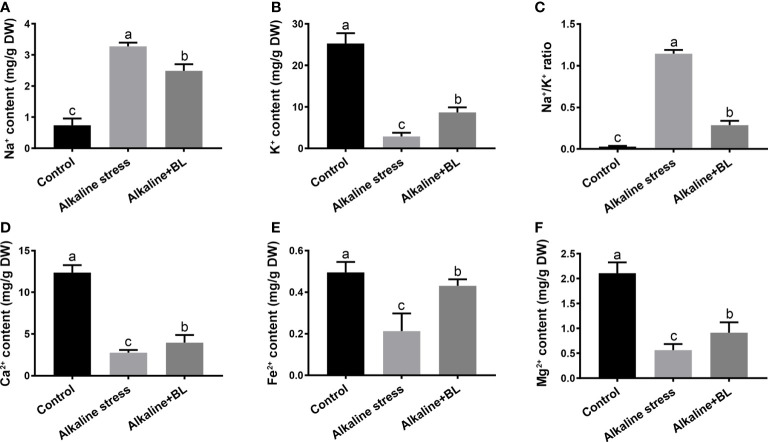
Effects of exogenous BL treatment on Na^+^ content **(A)**, K^+^ content **(B)**, and Na^+^/K^+^ ratio **(C)**, Ca^2+^ content **(D)**, Fe^2+^ content **(E)** and Mg^2+^ content **(F)** under alkaline stress. The data represent the mean ± SD of three biological replicates. Different lowercase letters indicate significant differences according to Fisher’s least significant difference (*P* < 0.05).

The mineral elements including micronutrients and macronutrients of apple seedlings were measured. The content of Na^+^ was significantly under alkaline stress but decreased when exogenous BL was applied (**Figure 7A**). In contrast to Na^+^, the content of K^+^ was sharply decreased under alkaline stress but increased when exogenous BL was applied (**Figure 7B**). The Na^+^/K^+^ ratio is an important indicator of plant tolerance to alkaline stress and was also detected. As shown in **Figure 7C**, the Na^+^/K^+^ ratio was significantly increased under alkaline stress compared with that in the control group but decreased to extremely after application of exogenous BL. The variation tendency of Ca^2+^, Fe^2+^, and Mg^2+^ was similar to that of K^+^ (**Figure 7D-F**).

### Effects of exogenous BL on the expression levels of alkaline-related genes in roots under alkaline stress

**Figure 8:**
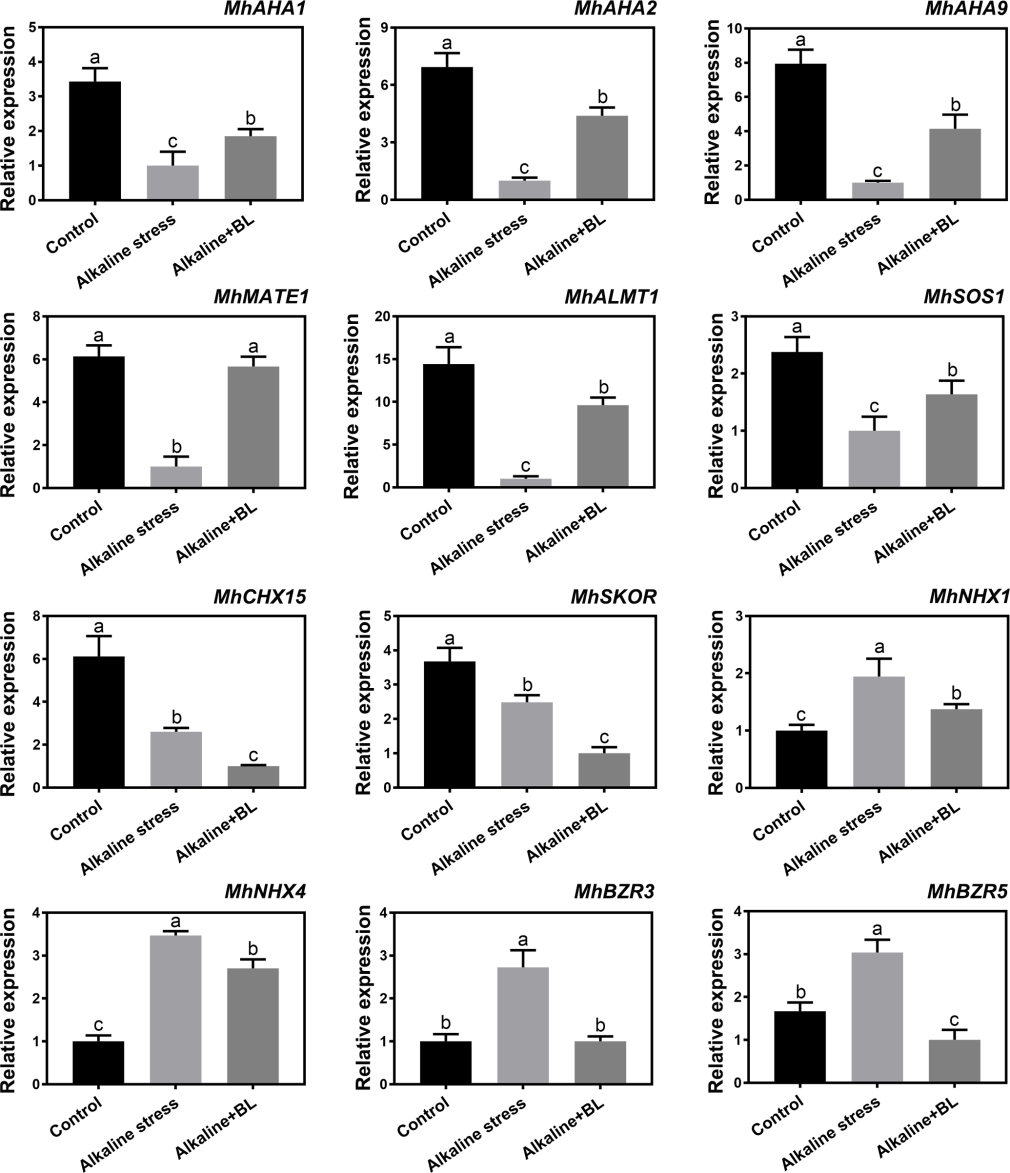
The expression of the 12 candidate genes (*MhAHA1*, *MhAHA2*, *MhAHA9*, *MhMATE1*, *MhALMT1*, *MhSOS1*, *MhCHX15*, *MhSKOR*, *MhNHX1*, *MhNHX4*, *MhBZR3* and *MhBZR5*) in roots under alkaline stress and exogenous BL treatment for 15 days. The data represent the mean ± SD of biological replicates. Different lowercase letters indicate significant differences according to Fisher’s least significant difference (*P* < 0.05).

As shown in **Figure 8**, the expression levels of 12 candidate genes, which were screened from RNA-Seq data (NCBI number: PRJNA588566) under alkaline stress, were detected under alkaline and exogenous BL treatment. These genes were divided into five categories. First, the three H^+^ transporter genes, namely, *MhAHA1*, *MhAHA2*, and *MhAHA9* had significantly decreased expression level under alkaline stress but significantly increased expression level when exogenous BL was applied. Second, the expression of organic acid transport genes, including *MhMATE1* and *MhALMT1*, were significantly decreased by alkaline stress and increased under exogenous BL treatment in the roots. Third, for Na^+^ transporter genes including *MhSOS1* and *MhCHX15*, the expression of *MhCHX15* showed a decreasing tendency under alkaline stress and exogenous BL treatment; however, the expression of *MhSOS1* was significantly increased after the application of exogenous BL. Fourth, for K^+^ transporter genes, a decreasing tendency variation was observed for *MhSKOR* under alkaline stress and exogenous BL treatment. *MhNHX1* and *MhNHX4* had significantly increased expression under alkaline stress but had significantly decreased expression when exogenous BL was applied. Finally, the expression of two selected transcription factors, namely, *MhBZR3* and *MhBZR5*, significantly changed under alkaline and exogenous BL treatment. For both of them, the expression level was increased significantly under alkaline stress, while decreased dramatically when exogenous BL was applied. These results suggested that exogenous BL responded to alkaline stress by regulating the expression of alkaline-related genes and *MhBZRs*.

## Discussion

Soil salinization seriously restricts the development of the global fruit industry (Hu et al., 2016). *M. hupehensis* is one of the most popular rootstocks in apple cultivation, but it is severely affected by saline-alkali stress (Su et al., 2020). Saline-alkali stress includes salt stress and alkaline stress. Alkaline stress causes more serious damage to plants than salt stress because it also induces high pH stress except for ion toxicity, osmotic stress, and oxidative damage (Yang and Guo, 2018; Fan et al., 2021). Nevertheless, most studies focus on the mechanism of plants in response to salt stress and on how to alleviate salt stress damage, ignoring the occurrence of salt stress is usually accompanied by alkaline stress in soil (Liang et al., 2017; Christian et al., 2018). Therefore, this study mainly focuses on the response of apple seedlings to alkaline stress.

Exogenous application of plant growth regulators is one of the effective methods to alleviate abiotic stress for hormones play important roles in plant growth and development and environmental stress response (Dong et al., 2017; Wu et al., 2017). Our previous study reported that exogenous BL could alleviate the salt stress of apple rootstock by regulating the transcription of NHX-type Na^+^ (K^+^)/H^+^ antiporters (Su et al., 2020). In the present study, we mainly focused on the root phenotype under alkaline stress. The roots were seriously damaged by alkaline stress with significantly short root length and less fibrous root number. Exogenous BL reduced the damage and partly recovered the root phenotype (**Figure 3**). Yang and Guo (2018) reported that the main damage to roots under alkaline stress was caused by high pH stress, which affected the roots absorption of nutrient elements and led to a series of symptoms related to nutrient deficiency. Ca is an important secondary messenger, maintaining its concentration in the cytoplasm can contribute to the regulation of plant signaling-transduction pathways under alkaline stress (Ding et al., 2010). Mg also has numerous positive effects on plant development (Zirek and Uzal, 2020). Fe is involved in chlorophyll synthesis and essential for maintaining the structure and function of chloroplasts (Casiraghi et al., 2020). Our results showed that the Ca^2+^, Fe^2+^ and Mg^2+^ contents were significantly decreased under alkaline stress, but increased when exogenous BL was applied (**Figure 7**). Hence, exogenous BL could promote Ca^2+^, Fe^2+^ and Mg^2+^ absorption, and the increase of the Fe^2+^ content would be the cause of the protection of chlorophyll and photosynthesis under alkaline stress (**Figure 2**). Previous study also reported that the application of BL promoted Ca^2+^ and Mg^2+^ absorption in soybean plants under normal conditions (Alam et al., 2019). The precipitation of nutrient elements could be attributed mainly to increased rhizosphere pH under alkaline stress (Veremeichik et al., 2021). Our staining results showed that the rhizosphere pH of apple roots significantly increased under alkaline stress but decreased after the application of exogenous BL (**Figure 4A**). However, the rhizosphere pH value under normal conditions was not affected by exogenous BL in cucumber (Wang et al., 2012). Our results suggested that the influence of exogenous BL to rhizosphere pH existed in the presence of alkaline stress. Root exudation of organic acids and H^+^-ATPase are the main responses of plants to high pH stress (Ghassemi-Golezani and Abdoli, 2021). On the one hand, we found that exogenous BL could significantly induce the expression of *MhALMT1* and *MhMATE1*, which are important genes involved in the transport of malic acid and citric acid. Moreover, the contents of malic acid and citric acid significantly decreased in the roots after exogenous BL was applied (**Figure 4** and **Figure 8**). These results indicated that exogenous BL could enhance the excretion of malic acid and citric acid outside of the roots in response to high pH stress. On the other hand, the H^+^-ATP enzyme in the plasma membrane acidified the pH environment in the roots through the external pump of H^+^ and improved the plants’ response to high pH stress (Sukhov et al., 2016; Zhao S. et al. (2016)). In this study, qPCR results also showed that exogenous BL could significantly induce the expression of *MhAHAs* (*MhAHA1*, *MhAHA2*, and *MhAHA9*), which encode the synthesis of H^+^-ATP enzyme (**Figure 8**). Taken together, our results indicated that exogenous BL could decrease the rhizosphere pH and promote Ca^2+^ and Mg^2+^ absorption by regulating malic acid and citric acid contents and increasing H^+^ excretion under alkaline stress.

Alkaline stress caused substantial accumulation of ROS in plants, resulting in oxidative damage (Sharma et al., 2016). Previous studies reported that salt stress could increase the contents of O_2_·^−^ and H_2_O_2_, whereas exogenous BL could alleviate this damage in leaves (Su et al., 2020). The present study focused on roots and found that the ROS level and MDA contents were significantly increased by alkaline stress and then decreased after the application of exogenous BL in the roots (**Figure 5**). SOD, POD and CAT are three major antioxidant enzymes (Tan et al., 2012; Abdelaal et al., 2018). They increased the resistance of sorghum roots to oxidative damage caused by heavy metal stress (Yilmaz et al., 2017). SOD is responsible for O_2_·^−^ clearance, while POD and CAT are responsible for H_2_O_2_ clearance in plant antioxidant systems. In this study, the results demonstrated that exogenous BL induced the activities of POD and CAT, but exerted no significant activity change in SOD to mitigate ROS in the roots (**Figure 5**). In addition, the O_2_·^−^ level had no significant change, but suffered severe H_2_O_2_ damage in leaves under alkaline stress. When exogenous BL was applied, the H_2_O_2_ content was substantially decreased (**Figure S1A**). Moreover, the POD and CAT activities were significantly repressed under alkaline stress but increased in leaves after BL was applied, while the SOD activity had no significantly change both in leaves and roots. Therefore, we speculated that exogenous BL could eliminate H_2_O_2_ through increasing the activities of POD and CAT, with no remarkably effect on SOD activity and O_2_·^−^ scavenging both in leaves and roots. The AsA-GSH cycle is an important non-enzymatic antioxidant protection system in plants, and its function on ROS scavenging is mainly through the combination of the antioxidants AsA, GSH and the key enzyme APX (Wang et al., 2019; Li et al., 2022). For normal cell functioning, exogenous BL supplementation is useful in maintaining the GSH/GSSG and AsA/DHA ratios (Batth et al., 2017; Alam et al., 2019). In this study, the change of APX activity was similar with POD and CAT, which was decreased under alkaline stress, but increased under exogenous BL treatment. The ratios of GSH/GSSG and AsA/DHA showed a significant decline under salt stress; nevertheless, application of exogenous BL improved productions of GSH and AsA, which transformed more GSSG and DHA to its reduced form and generate a reduced redox homeostatic environment (**Figure 5** and **Figure S2**). Taken together, our study concluded that exogenous BL has the potential to improving the activities of the antioxidant enzymes and modulate the AsA-GSH cycle to a redox state that plays a fundamental role in alkaline stress tolerance of apple plants.

Osmotic stress is a direct damage to plants caused by alkaline stress (Wu et al., 2017). Our results showed that electrolyte leakage was induced significantly under alkaline stress but decreased after exogenous BL treatment (**Figure 6**). Previous studies reported that the accumulation of substances such as proline and soluble sugars is a common defense mechanism of plants under osmotic stress (Liang et al., 2017; Zhang et al., 2017). Our data indicated that exogenous BL could protect apple seedlings from osmotic stress by accumulating proline (**Figure 6**).

Another major damage caused by alkaline stress is ion toxicity because large amounts of Na^+^ entering cells can lead to cation imbalance (Lv et al., 2013). Regulating Na^+^/K^+^ in the cytoplasm is one of the core mechanisms of plants in response to saline-alkali stress (Wang X. et al., 2015; Azhar et al., 2017). In the present study, the Na^+^/K^+^ ratio increased significantly under alkaline stress; after exogenous BL treatment, the K^+^ content increased and the Na^+^ content was inhibited (**Figure 7**). The SOS pathway is one of the classical responses of plants to alkaline stress, and SOS1 in the plasma membrane alleviates the harmful effects of alkaline stress by discharging Na^+^ from the cells (Fan et al., 2019; Gupta et al., 2021). Our results indicated that the expression of *MhSOS1* was significantly induced after exogenous BL application, resulting in reduced Na^+^ content (**Figure 7** and **Figure 8**). In the regulation of K^+^, the SKOR family in the plasma membrane functions in the efflux of potassium ions outside the cells (Zheng et al., 2020). NHX1-4 in tonoplast is responsible for K^+^ compartmentalization between the cytosol and the vacuole (Barragan et al., 2012; Li Y. et al. (2021); Solis et al., 2022). Exogenous BL treatment significantly decreased the levels of *MhSKOR*, *MhNHX1*, and *MhNHX4* (**Figure 8**). Hence, exogenous BL could inhibit K^+^ expel and compartmentalization, resulting in more K^+^ concentration in the cytoplasm to maintain relatively stable Na^+^/K^+^ ratio. In a word, the ion toxicity caused by alkaline stress could be alleviated by exogenous BL through enhancing Na^+^ efflux and inhibiting K^+^ expel and vacuole compartmentalization.

The expression of *MhBZRs*, as the key transcription factor in the BR signaling pathway, was also detected in the current study. The results indicated that the expression levels of *MhBZR3* and *MhBZR5* were significantly induced by alkaline stress and inhibited after exogenous BL was applied (**Figure 8**). This variation tendency was similar to that of K^+^ transport genes (*MhNHX1* and *MhNHX4*) and opposite to that of *MhAHAs*, *MhSOS1*, *MhMATE1*, and *MhALMT1*. BR regulates a variety of biological processes mainly through the key transcription factors (BZRs) in its signal transduction pathway, while the *BZR* family transcription factors can directly regulate gene expression to participate in biological reactions (Sun et al., 2022). Recently, it was found that MaBZR1, MaBZR2, and MaBZR3 could bind specifically to the promoters of ethylene biosynthesis-related genes, thus reducing ethylene synthesis, and then inhibit the ripening of banana fruit (Guo et al., 2019). In maize, *ZmBZR* genes responds positively to salt stress but negatively to high temperature stress (Manoli et al., 2018). Therefore, *BZRs* expression patterns differed among different species or within the same species under different stresses. Previous studies showed that MhBZR1 and MhBZR2 can bind to the promoter of *MhSOS1* or *MhNHX4-1* and inhibit their transcription, respectively (Sze and Chanroj, 2018; Fan et al., 2019; Su et al., 2020). Future research will focus on the regulatory relationship between BR-signaling transduction pathway genes and alkaline-related genes.

## Conclusion

Our study explored that exogenous BL could effectively improve the tolerance of apple plants on alkaline stress. Exogenous BL could decrease the rhizosphere pH by regulating malic acid and citric acid contents and increasing H^+^ excretion, reduce oxidative damage through increasing the activities of antioxidant enzymes and the antioxidants contents, regulate osmotic balance by accumulating proline, and alleviate ion toxicity through enhancing Na^+^ efflux and inhibiting K^+^ expel and vacuole compartmentalization. Overall, the application of exogenous BL mitigated the alkaline toxicity in apple plants and thus it can be applied to other plants as well. Such a sustainable approach can be used to achieve enhanced fruit production under saline-alkali soils.

## Data availability statement

The raw data supporting the conclusions of this article will be made available by the authors, without undue reservation.

## Author contributions

XZ and CW planned and designed the research. ZS, YZ, CX, LH, XZ, YT, CM, XL, and CW performed experiments, XZ, YT, CM, XL, and CW performed experiments, conducted fieldwork, analyzed data etc. XZ, YZ, ZS, and CW wrote the manuscript. All authors contributed to the article and approved the submitted version.

## Funding

This work was supported by the National Natural Science Foundation of China (32172542, 32102351), Breeding Plan of Shandong Provincial Qingchuang Research Team (2019), Funds for Modern Agricultural Industry Technology System in Shandong Province, China (SDAIT-06-06).

## Conflict of interest

The authors declare that the research was conducted in the absence of any commercial or financial relationships that could be construed as a potential conflict of interest.

## Publisher’s note

All claims expressed in this article are solely those of the authors and do not necessarily represent those of their affiliated organizations, or those of the publisher, the editors and the reviewers. Any product that may be evaluated in this article, or claim that may be made by its manufacturer, is not guaranteed or endorsed by the publisher.

## References

[B1] Abdelaal K. Hafez Y. M. El-Afry M. M. Tantawy D. S. Alshaal T. (2018). Effect of some osmoregulators on photosynthesis, lipid peroxidation, antioxidative capacity and productivity of barley (Hordeum vulgare l.) under water deficit stress. Environ. Sci. pollut. Res. Int. 25, 30199–30211. doi: 10.1007/s11356-018-3023-x 30155630

[B2] Ahmad P. Abdel Latef A. A. Hashem A. Abd Allah E. F. Gucel S. Tran L. (2016). Nitric oxide mitigates salt stress by regulating levels of osmolytes antioxidant enzymes in chickpea. Plant Sci. 7. doi: 10.3389/fpls.2016.00347 PMC4814448 27066020

[B3] Alam P. Albalawi T. H. Altalayan F. H. Bakht M. A. Ahanger M. A. Raja V.. (2019). 24-epibrassinolide (EBR) confers tolerance against NaCl stress in soybean plants by up-regulating antioxidant system, ascorbate-glutathione cycle, and glyoxalase system. Biomolecules 9 (11), 640. doi: 10.3390/biom9110640 31652728 PMC6920941

[B4] An J. P. Yao J. F. Xu R. R. You C. X. Wang X. F. Hao Y. J. (2018). An apple NAC transcription factor enhances salt stress tolerance by modulating the ethylene response. Physiol. Plant. 164, 279–289. doi: 10.1111/ppl.12724 29527680

[B5] Azhar N. Su N. Shabala L. Shabala S. (2017). Exogenously applied 24-epibrassinolide (EBL) ameliorates detrimental effects of salinity by reducing k+ efflux *via* depolarization-activated k+ channels. Plant Cell Physiol. 58 (4), 802–810. doi: 10.1093/pcp/pcx026 28340062

[B6] Barragan V. Leidi E. O. Andres Z. Rubio L. Luca A. D. Fernandez J. A.. (2012). Ion exchangers NHX1 and NHX2 mediate active potassium uptake into vacuoles to regulate cell turgor and stomatal function in arabidopsis. Plant Cell. 24 (3), 1127–1142. doi: 10.1105/tpc.111.095273 22438021 PMC3336136

[B7] Batth R. Singh K. Kumari S. Mustafiz A. (2017). Transcript profiling reveals the presence of abiotic stress and developmental stage specific ascorbate oxidase genes in plants. Front. Plant Sci. 8. doi: 10.3389/fpls.2017.00198 PMC5314155 28261251

[B8] Casiraghi F. M. Landi M. Donnini S. Borlotti A. Vigani G. (2020). Modulation of photorespiration and nitrogen recycling in fe-deficient cucumber leaves. Plant Physiol. Biochem. 154, 142–150. doi: 10.1016/j.plaphy.2020.05.032 32559518

[B9] Chen Z. Zhao P. Miao Z. Qi G. Wang Z. Yuan Y.. (2019). SULTR3s function in chloroplast sulfate uptake and affect ABA biosynthesis and the stress response. Plant Physiol. 180, 593–604. doi: 10.1104/pp.18.01439 30837346 PMC6501079

[B10] Christian Z. Christoph-Martin G. Karl-Josef D. (2018). Salinity and crop yield. Plant Biol. 21, 31–38. doi: 10.1111/plb.12884 30059606

[B11] Ding F. Chen M. Sui N. Wang B. (2010). Ca2+ signifificantly enhanced development and salt-secretion rate of salt glands of limonium bicolor under NaCl treatment. S. Afr. J. Bot. 76, 95–101. doi: 10.1016/j.sajb.2009.09.001

[B12] Dong Y. Wang W. Hu G. Chen W. Zhuge Y. Wang Z.. (2017). Role of exogenous 24-epibrassinolide in enhancing the salt tolerance of wheat seedlings. J. Soil Sci. Plant Nutt. 17 (3), 554–569. doi: 10.4067/S0718-95162017000300001

[B13] Fan Y. Lu X. Chen X. Wang J. Wang D. Wang S.. (2021). Cotton transcriptome analysis reveals novel biological pathways that eliminate reactive oxygen species (ROS) under sodium bicarbonate (NaHCO3) alkaline stress. Genomics 113, 1157–1169. doi: 10.1016/j.ygeno.2021.02.022 33689783

[B14] Fan Y. Yin X. Xie Q. Xia Y. Wang Z. Song J.. (2019). Co-Expression of SpSOS1 and SpAHA1 in transgenic arabidopsis plants improves salinity tolerance. BMC. Plant Biol. 19, 1–13. doi: 10.1186/s12870-019-1680-7 30764771 PMC6376693

[B15] Ghassemi-Golezani K. Abdoli S. (2021). Improving ATPase and PPase activities, nutrient uptake and growth of salt stressed ajowan plants by salicylic acid and iron-oxide nanoparticles. Plant Cell Rep. 40 (1), 1–15. doi: 10.1007/s00299-020-02652-7 33403499

[B16] Gong X. Shi S. Dou F. Song Y. Ma F. (2017). Exogenous melatonin alleviates alkaline stress in malus hupehensis rehd. by regulating the biosynthesis of polyamines. Molecules 22 (9), 1542. doi: 10.3390/molecules22091542 28902159 PMC6151414

[B17] Guo Y. F. Shan W. Liang S. M. Wu C. J. Wei W. Chen J. Y.. (2019). MaBZR1/2 act as transcriptional repressors of ethylene biosynthetic genes in banana fruit. Physiol. Plant. 165 (3), 555–568. doi: 10.1111/ppl.12750 29704245

[B18] Gupta B. K. Sahoo K. K. Anwar K. Nongpiur R. C. Deshmukh R. Pareek A.. (2021). Silicon nutrition stimulates salt-overly sensitive (SOS) pathway to enhance salinity stress tolerance and yield in rice. Plant Physiol. Biochem. 166, 593–604. doi: 10.1016/j.plaphy.2021.06.010 34186283

[B19] Hu Y. Xia S. Su Y. Wang H. Luo W. Su S.. (2016). Brassinolide increases potato root growth *in vitro* in a dose-dependent way and alleviates salinity stress. Biomed. Res. Int. 2016, 8231873. doi: 10.1155/2016/8231873 27803931 PMC5075642

[B20] Hu L. Zhou K. Li Y. Chen X. Liu B. Li C.. (2018). Exogenous myoinositol alleviates salinity-induced stress in malus hupehensis rehd. plant physiol. Biochem 133, 116–126. doi: 10.1016/j.plaphy.2018.10.037 30399545

[B21] Jia C. Zhao S. Bao T. Zhao P. Peng K. Guo Q.. (2021). Tomato BZR/BES transcription factor SIBZR1 positively regulates BR signaling and salt stress tolerance in tomato and arabidopsis. Plant Sci. 302, 110719. doi: 10.1016/j.plantsci.2020.110719 33288025

[B22] Ji Y. Zhang P. Xing Y. Jia L. Zhang Y. Jia T.. (2019). Effect of 1α, 25-dihydroxyvitamin D3 on the osteogenic differentiation of human periodontal ligament stem cells and the underlying regulatory mechanism. Int. J. Mol. Med. 43 (1), 167–176. doi: 10.3892/ijmm.2018.3947 30365053 PMC6257868

[B23] Liang W. Ma X. Wan P. Liu L. (2017). Plant salt-tolerance mechanism: A review. Biochem. Bioph. Res. Co. 495, 286–291. doi: 10.1016/j.bbrc.2017.11.043 29128358

[B24] Li B. Fu Y. Li X. Yin H. Xi Z. (2022). Brassinosteroids alleviate cadmium phytotoxicity by minimizing oxidative stress in grape seedlings: Toward regulating the ascorbate-glutathione cycle. Sci. Hortic. 299, 111002. doi: 10.1016/j.scienta.2022.111002

[B25] Li T. Li Y. Sun Z. Xi X. Sha G. Ma C.. (2021). Resveratrol alleviates the KCl salinity stress of malus hupehensis rhed. Front. Plant Sci. 12. doi: 10.3389/fpls.2021.650485 PMC8149799 34054896

[B26] Liu N. Jin Z. Wang S. Gong B. Wen D. Wang X.. (2015). Sodic alkaline stress mitigation with exogenous melatonin involves reactive oxygen metabolism and ion homeostasis in tomato. Sci. Hortic. 181, 18–25. doi: 10.1016/j.scienta.2014.10.049

[B27] Li H. Wang L. Luo Y. (2018). Composition analysis by UPLC-PDA-ESI (–)-HRMS and antioxidant activity using saccharomyces cerevisiae model of herbal teas and green teas from hainan. Molecules 23 (10), 2550. doi: 10.3390/molecules23102550 30301226 PMC6222971

[B28] Li Y. Wu Y. Liao W. Hu L. Dawuda M. M. Jin X.. (2020). Nitric oxide is involved in the brassinolide-induced adventitious root development in cucumber. BMC Plant Biol. 20 (1), 1–12. doi: 10.1186/s12870-020-2320-y 32138654 PMC7059714

[B29] Li J. Yang P. Xie J. Yu J. (2015). Effects of 24-epibrassinolide on growth and antioxidant enzymes system in pepper roots under chilling stress. J. Nucl. Agric. Sci. 29, 1001–1008. doi: 10.11869/j.issn.100-8551.2015.05.1001

[B30] Li Y. Zheng X. Tian Y. Ma C. Wang C. (2021). Comparative transcriptome analysis of NaCl and KCl stress response in malus hupehensis rehd. provide insight into the regulation involved in na+ and k+ homeostasis. Plant Physiol. Biochem. 164 (2), 101–114. doi: 10.1016/j.plaphy.2021.04.022 33975146

[B31] Lv B. Li X. Ma H. Sun Y. Wei L. Jiang C.. (2013). Differences in growth and physiology of rice in response to different saline-alkaline stress factors. Agron. J. 105 (4), 1119–1128. doi: 10.2134/agronj2013.0017

[B32] Ma C. Liang B. Chang B. Yan J. Liu L. Wang Y.. (2019). Transcriptome profiling of anthocyanin biosynthesis in the peel of 'Granny smith' apples (Malus domestica) after bag removal. BMC Genomics 20, 1–18. doi: 10.1186/s12864-019-5730-1 31072309 PMC6507055

[B33] Manoli A. Trevisan S. Quaggiotti S. Varotto S. (2018). Identification and characterization of the BZR transcription factor family and its expression in response to abiotic stresses in zea mays l. Plant Growth Regul. 84 (3), 423–436. doi: 10.1007/s10725-017-0350-8

[B34] Mao K. Dong Q. Li C. Liu C. Ma F. (2017). Genome wide identification and characterization of apple bHLH transcription factors and expression analysis in response to drought and salt stress. Front. Plant Sci. 8. doi: 10.3389/fpls.2017.00480 PMC5387082 28443104

[B35] Nawaz F. Naeem M. Zulfiqar B. Akram A. Ashraf M. Y. Raheel M.. (2017). Understanding brassinosteroid-regulated mechanisms to improve stress tolerance in plants: a critical review. Environ. Sci. Pollut. R. 24 (19), 15959–15975. doi: 10.1007/s11356-017-9163-6 28540554

[B36] Negi P. Mishra S. Ganapathi T. R. Srivastava A. K. (2021). Regulatory short RNAs: A decade's tale for manipulating salt tolerance in plants. Physiol. Plant. 173, 1535–1555. doi: 10.1111/ppl.13492 34227692

[B37] Ryu H. Cho Y. G. (2015). Plant hormones in salt stress tolerance. J. Integr. Plant Biol. 58, 147–155. doi: 10.1007/s12374-015-0103-z

[B38] Shahzad B. Tanveer M. Che Z. Rehman A. Cheema S. A. Sharma A.. (2018). Role of 24-epibrassinolide (EBL) in mediating heavy metal and pesticide induced oxidative stress in plants: A review. Ecotox. Environ. Safe. 147, 935–944. doi: 10.1016/j.ecoenv.2017.09.066 29029379

[B39] Sharma I. Bhardwaj R. Pati P. K. (2013). Stress modulation response of 24-epibrassinolide against imidacloprid in an elite indica rice variety pusa basmati-1. Pestic. Biochem. Phys. 105 (2), 144–153. doi: 10.1016/j.pestbp.2013.01.004

[B40] Sharma P. Kumar A. Bhardwaj R. (2016). Plant steroidal hormone epibrassinolide regulate-heavy metal stress tolerance in oryza sativa l. by modulating antioxidant defense expression. Environ. Exp. Bot. 122, 1–9. doi: 10.1016/j.envexpbot.2015.08.005

[B41] Solis C. A. Yong M. T. Zhou M. Venkataraman G. Shabala L. Holford P.. (2022). Evolutionary significance of NHX family and NHX1 in salinity stress adaptation in the genus oryza. int. J. Mol. Sci. 23 (4), 2092. doi: 10.3390/ijms23042092 PMC8879705 35216206

[B42] Song M. Chen F. F. Li Y. H. Zhang L. Wang F. Qin R. R.. (2018). Trimetazidine restores the positive adaptation to exercise training by mitigating statin-induced skeletal muscle injury. J. Cachexia. Sarcopeni. 9 (1), 106–118. doi: 10.1002/jcsm.12250 PMC5803604 29152896

[B43] Sukhov V. Surova L. Morozova E. Sherstneva O. Vodeneev V. (2016). Changes in h+-ATP synthase activity, proton electrochemical gradient, and pH in pea chloroplast can be connected with variation potential. Front. Plant Sci. 7. doi: 10.3389/fpls.2016.01092 PMC4956672 27499760

[B44] Sun Z. Guo D. Lv Z. Bian C. Ma C. Liu X.. (2022). Brassinolide alleviates fe deficiency-induced stress by regulating the fe absorption mechanism in malus hupehensis rehd. Plant Cell Rep. 41 (9), 1863–1874. doi: 10.1007/s00299-022-02897-4 35781542

[B45] Su Q. Zheng X. Tian Y. Wang C. (2020). Exogenous brassinolide alleviates salt stress in malus hupehensis rehd. by regulating the transcription of NHX-type na+ (K+)/H+ antiporters. Front. Plant Sci. 11. doi: 10.3389/fpls.2020.00038 PMC7016215 32117377

[B46] Sze H. Chanroj S. (2018). pH and ion homeostasis on plant endomembrane dynamics: insights from structural models and mutants of K+/H+ antiporters. Plant Physiol. 177, 875–895. doi: 10.1104/pp.18.00142 29691301 PMC6053008

[B47] Tan D. Hardeland R. Manchester L. C. Korkmaz A. Ma S. Rosales S. C.. (2012). Functional roles of melatonin in plants, and perspectives in nutritional and agricultural science. J. Exp. Bot. 63, 577–597. doi: 10.1093/jxb/err256 22016420

[B48] Tofighi C. Khavari-Nejad R. A. Najafi F. Razavi K. Rejali F. (2017). Responses of wheat plants to interactions of 24-epibrassinolide and glomus mosseae in saline condition. Physiol. Mol. Biol. Pla. 23 (3), 557–564. doi: 10.1007/s12298-017-0439-6 PMC5567700 28878494

[B49] Veremeichik G. N. Shkryl Y. N. Silantieva S. A. Gorpenchenko T. Y. Brodovskaya E. V. Yatsunskaya M. S.. (2021). Managing activity and Ca2+ dependence through mutation in the junction of the AtCPK1 coordinates the salt tolerance in transgenic tobacco plants. Plant Physiol. Biochem. 165, 104–113. doi: 10.1016/j.plaphy.2021.05.026 34034156

[B50] Wang X. Geng S. Ma Y. Shi D. Yang C. Wang H. (2015). Growth, photosynthesis, solute accumulation, and ion balance of tomato plant under sodium-or potassium-salt stress and alkali stress. Agron. J. 65 (2), 927–928. doi: 10.2134/agronj14.0344

[B51] Wang B. Li Y. Zhang W. H. (2012). Brassinosteroids are involved in response of cucumber (Cucumis sativus) to iron deficiency. Ann. Bot. 110 (3), 681–688. doi: 10.1093/aob/mcs126 22684685 PMC3400454

[B52] Wang T. Tohge T. Ivakov A. Mueller-Roeber B. Fernie A. R. Mutwil M.. (2015a). Salt-related MYB1 coordinates abscisic acid biosynthesis and signaling during salt stress in arabidopsis. Plant Physiol. 169, 1027–1041. doi: 10.1104/pp.15.00962 26243618 PMC4587467

[B53] Wang F. Zhang X. Yang Q. Zhao Q. (2019). Exogenous melatonin delays postharvest fruit senescence and maintains the quality of sweet cherries. Food Chem. 301, 125311. doi: 10.1016/j.foodchem.2019.125311 31398670

[B54] Wu J. Cao J. Su M. Feng G. Xu Y. Yi H. (2019). Genomewide comprehensive analysis of transcriptomes and small RNAs offers insights into the molecular mechanism of alkaline stress tolerance in a citrus rootstock. Hortic. Res. 6, 33–51. doi: 10.1038/s41438-018-0116-0 30854210 PMC6395741

[B55] Wu W. Zhang Q. Ervin E. Yang Z. Zhang X. (2017). Physiological mechanism of enhancing salt stress tolerance of perennial ryegrass by 24-epibrassinolide. Front. Plant Sci. 8. doi: 10.3389/fpls.2017.01017 PMC5474491 28674542

[B56] Xia L. Marquès-Bueno M. Bruce C. G. Karnik R. (2019). Unusual roles of secretory SNARE SYP132 in plasma membrane h+-ATPase traffic and vegetative plant growth. Plant Physiol. 180, 837–858. doi: 10.1111/ppl.13492 30926657 PMC6548232

[B57] Xiong M. Yu J. Wang J. Gao Q. Huang L. Chen C.. (2022). Brassinosteroids regulate rice seed germination through the BZR1-RAmy3D transcriptional module. Plant Physiol. 189 (1), 402–418. doi: 10.1093/plphys/kiac043 35139229 PMC9070845

[B58] Yang Y. Guo Y. (2018). Elucidating the molecular mechanisms mediating plant salt-stress responses. New Phytol. 217, 523–539. doi: 10.1111/nph.14920 29205383

[B59] Yilmaz S. H. Kaplan M. Yilmaz R. Temizgul S. (2017). Antioxidant enzyme response of sorghum plant upon exposure to aluminum, chromium and lead heavy metals. Turk. J. Biochem. 42 (4), 503–512. doi: 10.1515/tjb-2016-0112

[B60] Yuan L. Zhu S. Shu S. Sun J. Guo S. (2015). Regulation of 2, 4-epibrassinolide on mineral nutrient uptake and ion distribution in Ca(NO3)2 stressed cucumber plants. J. Plant Physiol. 188, 29–36. doi: 10.1016/j.jplph.2015.06.010 26398630

[B61] Zhang H. Liu X. Zhang R. Yuan H. Wang M. Yang H.. (2017). Root damage under alkaline stress is associated with reactive oxygen species accumulation in rice (Oryza sativa l.). Front. Plant Sci. 8. doi: 10.3389/fpls.2017.01580 PMC5596797 28943882

[B62] Zhan H. Nie X. Zhang T. Li S. Wang X. Du X.. (2019). Melatonin: a small molecule but important for salt stress tolerance in plants. Int. J. Mol. Sci. 20 (3), 709. doi: 10.3390/ijms20030709 30736409 PMC6387279

[B63] Zhao C. Jiang W. Omar Z. Liu X. Tang K. Nie W.. (2021). The LRXs-RALFs-FER module controls plant growth and salt stress responses by modulating multiple plant hormones. Natl. Sci. Rev. 8 (01), 40–55. doi: 10.1093/nsr/nwaa149 PMC8288382 34691553

[B64] Zhao Q. Ren Y. Wang Q. Yao Y. You C. Hao Y. (2016). Overexpression of MdbHLH104 gene enhances the tolerance to iron deficiency in apple. Plant Biotechnol. J. 14, 1633–1645. doi: 10.1111/pbi.12526 26801352 PMC5066684

[B65] Zhao S. Zhang M. Ma T. Wang Y. (2016). Phosphorylation of ARF2 relieves its repression of transcription of the k+ transporter gene HAK5 in response to low potassium stress. Plant Cell. 28, 3005–3019. doi: 10.1105/tpc.16.00684 27895227 PMC5240742

[B66] Zheng X. Chen H. Su Q. Wang C. Sha G. Ma C.. (2021). Resveratrol improves the iron deficiency adaptation of malus baccata seedlings by regulating iron absorption. BMC Plant Biol. 21, 433. doi: 10.21203/rs.3.rs-168975/v1 34556040 PMC8459475

[B67] Zheng X. Li Y. Xi X. Ma C. Sun Z. Yang X.. (2020). Exogenous strigolactones alleviate KCl stress by regulating photosynthesis, ROS migration and ion transport in malus hupehensis rehd. Plant Physiol. Biochem. 159, 113–122. doi: 10.1016/j.plaphy.2020.12.015 33359960

[B68] Zheng X. Zhou J. Tan D. Wang N. Wang L. Shan D.. (2017). Melatonin improves waterlogging tolerance of malus baccata (Linn.) borkh. seedlings by maintaining aerobic respiration, photosynthesis and ROS migration. Front. Plant Sci. 8. doi: 10.3389/fpls.2017.00483 PMC5380759 28424730

[B69] Zhu J. K. (2016). Abiotic stress signaling and responses in plants. Cell 167, 313–324. doi: 10.1016/j.cell.2016.08.029 27716505 PMC5104190

[B70] Zirek N. S. Uzal O. (2020). The developmental and metabolic effects of different magnesium dozes in pepper plants under salt stress. No. Bot. Horti. Agrobo. 48 (2), 967–977. doi: 10.15835/nbha48211943

