# Supplementary material for: Brassinolide improves the tolerance of Malus hupehensis to alkaline stress
Source: Front Plant Sci. 2022 Nov 24;13:1032646. doi: 10.3389/fpls.2022.1032646 (PMC9731795; doi:10.3389/fpls.2022.1032646)
Supplement: Supplementary file 1 [file DataSheet_1.docx]

**Supplementary Materials**

**Table S1** Primer sequences for qRT-PCR

| Primer name | Forward primer | Reverse primer |
| --- | --- | --- |
| *MhAHA1* | CCAGAGAAAACAAAAGAGAGTC | TTCACATTCACACCGAGATTG |
| *MhAHA2* | AAAGTGTGAAGAAAGAGAAAATGC | AGTTCATTTGCTTGACATTCTTT |
| *MhAHA9* | TTCATGGTCTTCAGCCACCT | TCACAACTGACTCGACGTGA |
| *MhMATE1* | ATTCCTGTTGTCTTGGTGTGCTT | GCCTTATCGTAGTCTTTTTTGGC |
| *MhALMT1* | TGATATCTGTGTCCGGTTTTCGAG | GAGAACAGTTTTGTATCCTTGGAG |
| *MhSOS1* | TACATCATTTCTGGTATATCTTGTG | CAAGATGAAAATTAAGGTATTAGCA |
| Mh*CHX15* | CCTCTTGGTACAGCATTGATAAAAA | GTTTGAACTTAATTTTGCAGCACA |
| *MhSKOR* | CATCCTGACAACTGGTGGTATCG | AAGTACCTCAGAGCAATCCGTTT |
| *MhNHX1* | GCTTATGCGTGGCTCTGTTTC | CGGGTCCTATGTTTGCCTCTG |
| *MhNHX4* | ACGAAACTCCTTTACTATACAGCCT | TGATACCACAGATAAGTGAGCATAG |
| *MhBZR3* | GAGGATGGCACCACCTACCG | CTGGGATTTCAACAAGAAAGCAAG |
| *MhBZR5* | GGGCAGCATTACCCTTTCCT | CGAGATCATCTGATACGCATTCC |
| *MhActin* | CTTCAATGTGCCTGCCATGTAT | AATTTCCCGTTCAGCAGTAGTG |


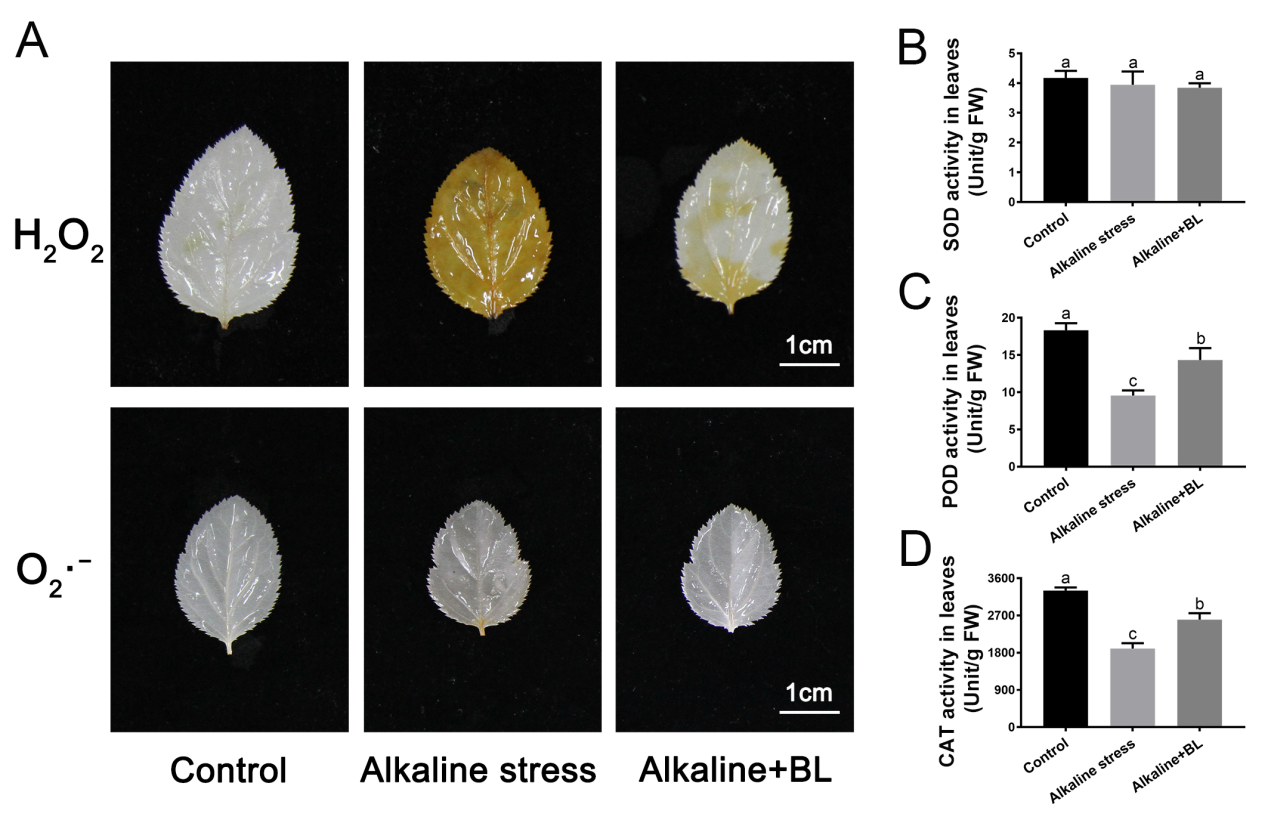


**Figure S1** Effects of exogenous BL on the oxidative damage and antioxidant enzyme activities of *M. hupehensis* seedlings leaves under alkaline stress. **(A)** Effects of alkaline stress and exogenous BL on the H_2_O_2_ and O_2_·^−^ levels in leaves. The scale bar represents 1.0 cm. Effects of exogenous BL treatment on SOD activity **(B)**, POD activity **(C)** and CAT activity **(D)** in leaves under alkaline stress. The data represent the mean ± SD of three biological replicates. Different lowercase letters indicate significant differences according to Fisher’s least significant difference (*P* < 0.05).


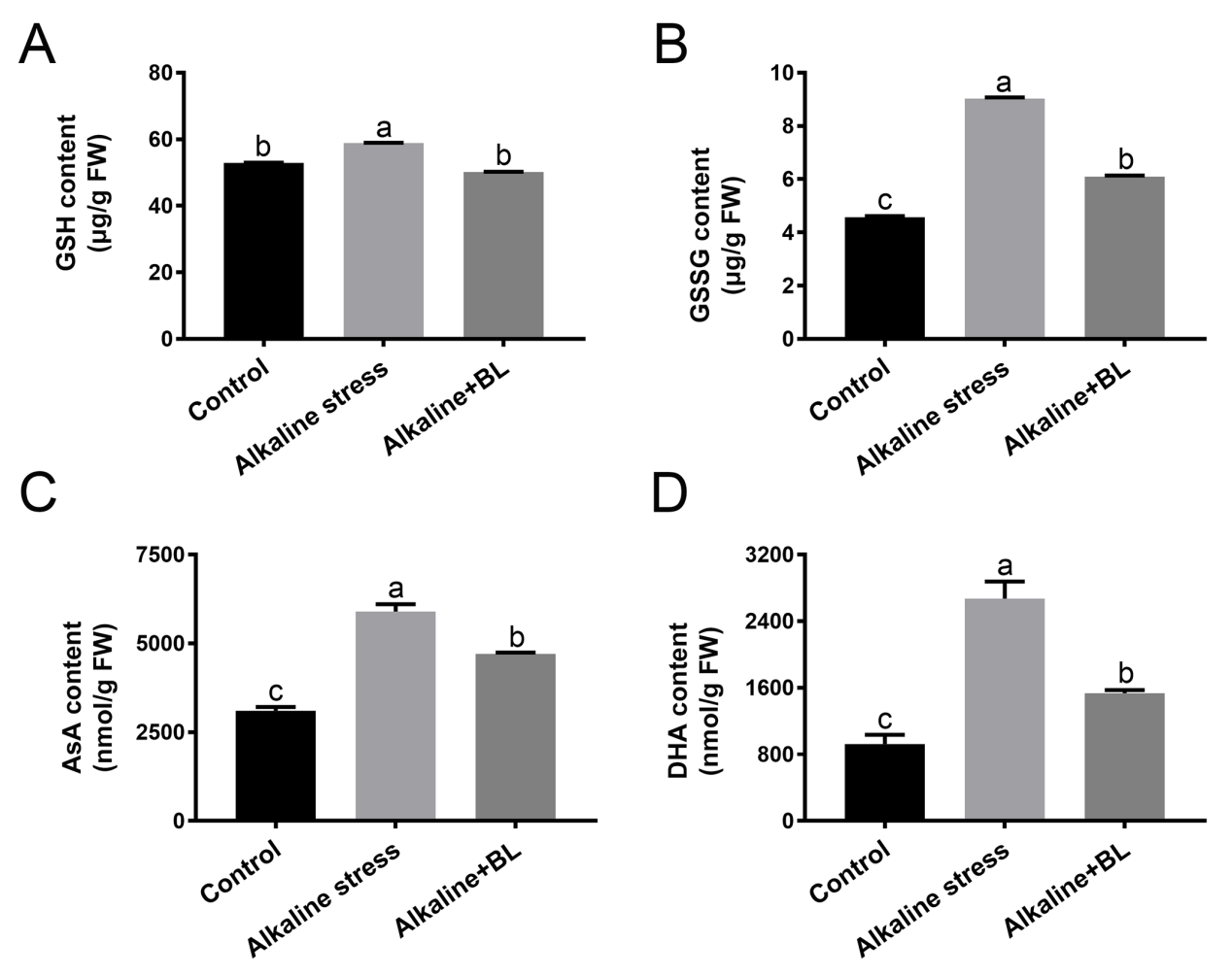


**Figure S2** Effects of exogenous BL on the antioxidants contents of *M. hupehensis* seedlings roots under alkaline stress. Effects of exogenous BL treatment on GSH content **(A)**, GSSG content **(B)**, AsA content **(C)** and DHA content **(D)** under alkaline stress. The data represent the mean ± SD of three biological replicates. Different lowercase letters indicate significant differences according to Fisher’s least significant difference (*P* < 0.05).
